# Antibiotic, Pesticide, and Microbial Contaminants of Honey: Human Health Hazards

**DOI:** 10.1100/2012/930849

**Published:** 2012-10-14

**Authors:** Noori Al-Waili, Khelod Salom, Ahmed Al-Ghamdi, Mohammad Javed Ansari

**Affiliations:** ^1^Waili Foundation for Science, Queens, NY 11418, USA; ^2^Department of Plant Protection, College of Food and Agricultural Sciences, King Saud University, Riyadh 11543, Saudi Arabia

## Abstract

Agricultural contamination with pesticides and antibiotics is a challenging problem that needs to be fully addressed. Bee products, such as honey, are widely consumed as food and medicine and their contamination may carry serious health hazards. Honey and other bee products are polluted by pesticides, heavy metals, bacteria and radioactive materials. Pesticide residues cause genetic mutations and cellular degradation and presence of antibiotics might increase resistant human or animal's pathogens. Many cases of infant botulisms have been attributed to contaminated honey. Honey may be very toxic when produced from certain plants. Ingestion of honey without knowing its source and safety might be problematic. Honey should be labeled to explore its origin, composition, and clear statement that it is free from contaminants. Honey that is not subjected for analysis and sterilization should not be used in infants, and should not be applied to wounds or used for medicinal purposes. This article reviews the extent and health impact of honey contamination and stresses on the introduction of a strict monitoring system and validation of acceptable minimal concentrations of pollutants or identifying maximum residue limits for bee products, in particular, honey.

## 1. Introduction 

History has revealed that humans had used bee products such as honey for thousands of years in all societies worldwide. Honey has been mentioned in the Talmud, the old and new testaments of the Bible, and the Holy Quran (1400 years ago). There is a large chapter (SORA) in Holey Quran named Bee (Al Nahl) and part of it says “ And thy LORD taught the bee to build its cells in hills, on trees and in men's habitations, then to eat of all the produce of the earth and find with skill the spacious paths of its LORD, there issues from within their bodies a drink of varying colors, wherein is healing for men, verily in this is a sign for those who give thought.” The Muslim prophet Mohammed even recommended the use of honey for the treatment of diarrhea [1]. Honey has been used to treat coughs and sore throats, infected leg ulcers, earaches, measles, eye diseases, and gastric ulcers [2–4]. Bee products are natural food products; they are rich in minerals, antioxidants, and simple sugars. Honey is known to be rich in both enzymatic and nonenzymatic antioxidants. Honey can also prevent deteriorative oxidation reactions in foods, such as the browning of fruit and vegetables and lipid oxidation in meat, as well as inhibit the growth of food borne pathogens and microorganisms that cause food spoilage [5, 6]. 

Others and we have found that honey has potential therapeutic properties in infections, wound healing, and cancer [7–11]. Therefore, in recent years, bee products have received renewed interest as an essential natural resource that can be employed in new therapies free from side effects that are often encountered with the use of synthetic chemical medicines [12]. However, the market competition on these products imposes extra conditions that can only be ensured by complying with quality assurance and certification protocols.

Bee products, including honey, are polluted via different sources of contamination (Table 1). Environmental contaminants include pesticides, heavy metals, bacteria, and radioactive materials. Pesticide residues have been shown to cause genetic mutations and cellular degradation. In addition to the public health problems, the presence of pesticides in bee products decreases its quality. According to European Union regulations, honey as a natural product must be free of chemicals [13]. Poisoning of bee pollinators is a serious adverse effect of insecticide use, which leads to a decrease in the insect population, reduction of honey yields, destruction of plant communities, presence of insecticide residues in food, and ultimately to a significant loss of a beekeepers' income. Basically, the main purposes for monitoring bee products are consumer health protection, international commercial competition, and better product quality. 

Antibiotics used in honey and other bee products production are usually those used in a veterinary setting, such as streptomycin, sulfonamide, and chloramphenicol. Obviously, beekeepers use antibiotics at relatively high doses to treat infections, or at low doses as “growth promoters.” Maximum residue limits (MRLs) have been established for most foods produced by animals treated with sulfonamides and tetracyclines. However, there are no MRLs for bee products such as honey. Honey is traded internationally and countries generally accept standards set by the Codex Alimentarius. Nevertheless, European countries, the US, Canada, Australia, and India have their own separate standards. Aberrantly, the extensive use of antibiotics leads to an accumulation of antibiotic residues in honey, thereby leading to decreased quality and difficulty in marketing [14]. Antibiotic residues have a relatively long half life and may have direct toxic effects on consumers [14]. Monitoring pesticide residues in honey, wax, and bees helps to assess the potential risk of these products to human health and provides data on the extent of pesticide treatments that have been used on the field crops surrounding the hives.

## 2. Pesticides and Honey Contamination

### 2.1. Pesticides

Pesticides are worldwide used in control of bee diseases and pests and in most instant their administration is uncontrolled and applied without approved protocols. The use of pesticides to protect crops is used to increase agricultural productivity. However, uncontrolled application may cause the contamination of environment, animal species, and human being. 

Pesticides residues include acaricides, organic acids, insecticides, fungicides, herbicides, and bactericides. Many of these contaminants are banned because of their well-documented health hazards such as carcinogenic effect on humans. Many toxic substances used to control varroatosis and ascospheriosis such as acaricides amitraz, celazole, bromopropylate, coumaphos, flumethrin, and taufluvalinate. The use of these chemicals inside beehives carries a risk of direct contamination of honey and other hive products [15]. Over 150 different pesticides have been found in colony samples [16]. The highest residues of pesticides are from varroacides that accumulate in beeswax, pollen, and bee bread and their residue levels increase from honey to pollen to beeswax [16–18]. 

MRLs of many contaminants have been set to levels as small as parts per billion. Different national regulations have established maximum concentrations of pesticide residues permitted in honey, but the lack of homogeneity causes problems in international marketing and trade. Germany, Italy, and Switzerland have set different MRLs for amitraz, bromopropylate, coumaphos, cyamizole, flumetrine, and fluvalinate [19]. The maximum limits of pesticide residues in honey are not included in the Codex Alimentarius [20]. The European Union legislation has regulated the MRLs for three amitraz, coumaphos, and cyamizole, which are 0.2, 0.1, and 1 mg·kg^−1^, respectively [21]. The US Environmental Protection Agency has established MRLs for amitraz (1 mg·kg^−1^), coumaphos (0.1 mg·kg^−1^), and fluvalinate (0.05 mg·kg^−1^) [22]. 

Most studies determine residues of acaricides that are used to control *Varroa jacobsoni* [23, 24]. The most often detected acaricides are bromopropylate, coumaphos, and fluvalinate. Few studies focused on pesticides used for crop protection and introduced into hives by contaminated bees and wax [25]. Furthermore, the fungicides are other important honey contaminants.

### 2.2. Honey Contamination with Pesticides

Honey can be contaminated from the environment and from beekeeping practice. A review has shown that honey could be contaminated from different pesticides [21]. It was found that the contamination of honey and other bee products with varroacides is more than ones originating from the environment. Because no MRLs have been established for pesticides in honey, it is hard to evaluate the contamination of honey with pesticides and the extent of possible damage to human health. Bogdanov reviewed different studies carried out on organochlorine pesticides residues in honey; the levels found in different countries differed considerably [19]. 

In India, a study was carried out to explore the extent of pesticide residue in honey produced in the various parts of Himachal Pradesh. It was found that HCH and its isomers were the most frequently detected followed by dichlor-diphenyl-trichlorethylene (DDT) and its isomers. Malathion's residue was found exceeding the MRLs (5 ppb) proposed by the Ministry of Commerce, Government of India. The results showed that honey from natural vegetation contained lesser residues [26]. In addition, levels and frequency of organophosphorus and carbamate pesticides were relatively higher in honey samples analyzed in India from 1993–1997 [27].

The majority of honey samples analyzed from Jordan during 1995 contained residues of organochlorine pesticides such as r-HCH, a-HCH, and lindane; with some contaminated with organophosphorus pesticides [28].

In Turkey, 24 organochlorine pesticide residues in 109 different honey samples collected from stores and open markets in Konya, Turkey, were analyzed by gas chromatography-electron capture detection. Aldrin, *cis*-chlordane, *trans*-chlordane, oxy-chlordane, 2,4(′)-DDE, and 4,4(′)-DDE were found in all honey samples. In the 55/109 samples, levels of organochlorine pesticide residues of oxy-chlordane were determined as higher than those of Turkish Alimentarius Codex MRLs. The study revealed that all of the honey samples are contaminated and most of these samples exceeded MRLs [29]. In same country, 32 pesticide residues and eight polybrominated diphenly ether congeners were measured in nine honey samples [30]. However, in another study, a number of 15 organophosphorus insecticides were investigated in 275 honey samples in 33 different cities of Turkey. When the limit of determination values was detected between 0.25 and 9.55 ng·g^−1^, no insecticide residue was detected in the samples analyzed [31]. An investigation of the naphthalene concentration in samples of contaminated honey consumed in Turkey was reported. Analysis of 100 samples of commercially available honey obtained from markets (53 samples) and street bazaars (47 samples) revealed that the mean naphthalene recovery from honey known to be contaminated with 1 microg/g was 80.4% [32]. 

In Spain, residues of thymol were found in honey collected from the beehives, ranged from 0.75 to 8.20 *μ*g·g^−1^ for Apilife Var [33]. Fifty samples of honey collected from local markets of Portugal and Spain during year 2002 were analyzed for various pesticides which included 42 organochlorine, carbamate, and organophosphorus [34]. It was found that Portuguese honeys were more contaminated than Spanish ones [34].

In France, a field survey was initiated in French apiaries in order to monitor the health of honey bee colonies. Beeswax samples were collected once a year over 2 years from a total of 125 honey bee colonies. Residues of 14 of the searched compounds (16 insecticides and acaricides and two fungicides) were found in samples; tau-fluvalinate, coumaphos, and endosulfan residues were the most frequently occurring residues. Beeswax contamination was the result of both in-hive acaricides treatments and environmental pollution [35].

Honey can be contaminated by fungicides used against pests in fruit trees and rape [36]. In Poland, different fungicides which included vinclozolin, iprodione, methyl thiophanate, captan, and difenoconazole were applied in cherry trees; the residue level of these fungicides were recovered from honey and pollen [36, 37]. In Switzerland, honey residues of the fungicides dithianon, pyrifenox, penconazol and cyproconazole which were applied in fruit trees in spring had been detected [38]. In Germany, it was found that carbendazim caused significant residues [39].

In US, 50 honey samples from Virginia were analyzed for the presence of fluvalinate and coumaphos residues. Samples were collected from both hives and bottled honey provided by beekeepers. Except for trace levels of coumaphos detected in three samples from hives and trace levels of fluvalinate found in one hive sample, no coumaphos or fluvalinate residues above the limit of quantification were detected in any of the samples. In addition, no residues were detected in any of the bottled honey samples [40].

Organic contaminants and polychlorinated biphenyl (PCB's), which originate from motor oil, coolants, and lubricants, are still present in the environment and can contaminate bees and their products. The quantities in honey are low while those in wax are high [41–43].

### 2.3. Health Impact of Pesticides

Systemic introduction of pesticides into nectar and pollen may have direct consequences for honey bee health and ultimately lead to pesticide contamination of honey-containing food. The effects of pesticides on human health are harmful based on the toxicity of the chemical and the length and magnitude of exposure [44]. Aberrantly, farm workers and their families have the greatest exposure to agricultural pesticides. Children are most susceptible and sensitive to pesticides due to their small size and underdevelopment. Importantly, the chemicals have the ability to bioaccumulate and biomagnify and can bio concentrate in the body over time.

Effect of exposure to pesticides ranges from mild skin irritation to birth defects, tumors, genetic changes, blood and nerve disorders, endocrine disruption, and even coma or death. Some pesticides, including Aldrin, chlordane, DDT, dihedron, endrin, heptachlor, hexachlorobenzene, mirex, and toxaphene, are considered persistent organic pollutants (POPs) [45]. POPs may compromise endocrine, reproductive, and immune systems. Many diseases such as cancer; neurobehavioral disorder infertility, and mutagenic effects might result from chronic exposure. Therefore, some POPs have been banned while others continue to be used [46, 47].

## 3. Antibiotics and Honey Contamination

### 3.1. Honey Contamination

Antibiotics are found in honey because they are used in apiculture for treatment of bacterial diseases. Antibiotic residues originate mostly from the environment and improper beekeeping practices. There are several international reports of antibiotic residues in honey samples. Oxytetracycline and chloramphenicol residues have been found above the regulatory standards in honey [48, 49]. Oxytetracycline is commonly used to treat European foulbrood disease and American foulbrood diseases caused by *Paenibacilus* (*Bacillus*) larvae and *Streptococcus pluton* bacteria, respectively. However, because of its widespread use, there are reports of tetracycline resistance in these bacteria. Other antibiotics such as erythromycin, lincomycin, monensin, streptomycin, and enrofloxacin are also used.

Fifty honey samples, collected from the Southern Marmara region of Turkey, were analyzed for erythromycin residues. Four of the honey samples were found to be contaminated with erythromycin residues at concentrations ranging from 50 to 1776 ng·kg^−1^. An erythromycin-fortified cake feeding assay was performed in a defined hive to test the transfer of erythromycin residue to the honey matrix; the residue level in the honey, 3 months after dosing, was approximately 28 ng·kg^−1^ [50].

In another study, samples of honey were then collected at intervals over a 41-week period after bee colonies were treated with 1.2 gram lincomycin hydrochloride per hive. Results showed that the highest mean concentration of lincomycin was 24 *μ*g^−1^ 3 days after treatment and a mean of 3.5 *μ*g^−1^ after 129 days. Importantly, lincomycin was persistent in the hive and detected in all over winter (290 days after dosing) samples of honey collected from both nonshook swarmed and shook swarmed colonies [51].

In China, five antibiotics compounds, tetracycline, oxytetracycline, doxycycline, chlortetracycline, and chloramphenicol, were successfully separated and determined in honey samples. The detection limits were 10 microg/L for chloramphenicol, 20 *μ*g/L for tetracycline, oxytetracycline, and doxycycline, and 40 *μ*g/L for chloramphenicol [52].

In India, high levels of antibiotics in honey exported from India to EU and US have been reported by Agricultural Processed Food Product Export Development Agency from 2005 onwards [53]. In 2006, about 14% samples were contaminated with tetracycline and in 2007-2008 about 28% samples were contaminated with same antibiotic. In 2009-2010, of the 362 honey samples tested, 29.2% samples had more than the prescribed limit of antibiotics. In 2000-2001, streptomycin was detected in 4/248, tetracycline in 2/72, and sulfonamides in 1/72 samples. Nectar and honey samples collected from bee hives during the peak flowering seasons of rubber (March to April) and banana (December to January) plantation crops in southern part of Tamil Nadu were analyzed for antibiotic residues. These samples showed 4–17 and 11–29 ng·kg^−1^ of streptomycin, 2–29 and 3–44 ng·kg^−1^ of ampicillin, and 17–34 and 26–48 ng/kg of kanamycin, respectively [54].

The use of antibiotics in beekeeping is illegal in some EU countries. However, there are no MRLs established for antibiotics in honey according to European Community regulations, which means that honey containing antibiotics residues are not permitted to be sold [55]. Some countries, like Switzerland, UK, and Belgium, have established action limits (level of antibiotics in honey beyond which the sample is deemed noncompliant) for antibiotics in honey, which generally lies between 0.01 to 0.05 mg/kg for each antibiotic group.

In Switzerland, a study involving 75 samples (34 originated from Asian countries) showed that 13 samples contained chloramphenicol residues [48]. 

In Greece, 251 honey samples produced across were analyzed by liquid chromatography to detect tetracycline-derived residues; 29% of the samples had tetracycline residues [49].

In France, tetracycline residues were detected in honey after a treatment in hives, indicating their persistence and diffusion into the apiary. These results showed that the tetracycline must be used with precaution in honey production [56].

In UK, a study aimed to assess oxytetracycline residue levels in honey after treatment of honeybee colonies with two methods of application in liquid sucrose and in powdered icing sugar. The samples of honey were extracted up to 12 weeks after treatment. It was demonstrated that the method of application of oxytetracycline in liquid form results in high residue levels in honey with residues of 3.7 mg/kg, eight weeks after application [57].

In Switzerland, bees occasionally collect nectar from meadows treated with the herbicide asulam. It was found that such honey is not only contaminated by asulam but also by its degradation product sulfanilamide. Hence, the use of herbicide causes the appearance of residues of an antibacterial active metabolite belonging to the category of sulfonamide drugs in food [58].

 In Granada and Almeria (two Spanish cities) residues of sarafloxacin, tylosin, sulfadimidine and sulfachlorpyridazine were found in two honey samples [59]. The fire blight on fruit trees is caused by *Erwinia amylovora*; streptomycin can control this bacteria. In Germany, it was found that honey was contaminated and 21% of 183 samples of honey contained streptomycin residues [60].

In Belgium, a migration test was set up to study whether sulfonamide-containing beeswax could lead to the contamination of honey. It was found that the higher the concentration of sulfamethazine doped in the wax, the higher was the concentration of sulfamethazine found in the honey [61].

Out of the 3855 honey samples tested, 1.7% samples were noncompliant with the EU standards; streptomycin, sulfonamides, tetracycline, chloramphenicol, nitrofurans, tylosin, and quinolones were detected in honey samples [62].

### 3.2. Health Impact of Antibiotics in Honey

Antibiotic residues in honey have become a major consumer concern. Some drugs have the potential to produce toxic reactions in consumers directly while some other is able to produce allergic or hypersensitivity reactions [63]. Lactam antibiotics cause cutaneous eruptions, dermatitis, gastrointestinal symptoms and anaphylaxis at very low doses [64]. 

Long-term effects of exposure to antibiotic residues include microbiological hazards, carcinogenicity, reproductive effects, and teratogenicity. Microbiological effects are one of the major health problems in human beings. Certain drugs like nitrofurans and nitroimidazoles can cause cancer in human being. Similarly, some drugs can produce reproductive and teratogenic effects at very low doses.

Antibiotic residues consumed along with food and honey can produce resistance in bacterial populations. Antibiotic resistance is a global public health problem and continues to be a challenging issue. The US Centers for Disease Control and Prevention (2000) has described antibiotic resistance as “one of the world's most pressing health problems,” because “the number of bacteria resistant to antibiotics has increased, and many bacterial infections are becoming resistant to the most commonly prescribed antibiotic treatments. The WHO has identified antibiotic resistance as “one of the three greatest threats to human health.” The primary cause is long-term exposure to antibiotics through their use as medicines in humans and animals, horticulture and for food preservation. The types of antibiotics used in animals are frequently similar to those used in humans.

In December 2003, workshop convened by the Food and Agriculture Organization of the United Nations, the World Organization for Animal Health and the WHO concluded that “there is clear evidence of adverse human health consequences due to resistant organisms resulting from nonhuman usage of antimicrobials. These consequences include infections that would not have otherwise occurred, increased frequency of treatment failures, and increased severity of infections.” 

More evidence documented an association between use of antibiotic agents in food animals and antibiotic resistance among bacteria isolated from humans. An outbreak of human nalidixic acid-resistant *Salmonella typhimurium* DT104 infection in Denmark was traced to a pig farm. Another outbreak of the same infection, reported in the UK, was traced to a dairy farm where fluoroquinolones had been used on the cattle a month before the outbreak. In the US, there was a marked increase in the proportion of domestically acquired *Campylobacter* infections that were fluoroquinolones resistant, following the first approved use of fluoroquinolones in food animals in 1995.

The WHO has recommended that antibiotics which are licensed in human medicine should not be used as growth promoters in livestock. Since then, studies from Denmark, Germany, and Italy have shown a significant reduction in vancomycin-resistant enterococcus isolations from poultry and poultry-derived food products. Some European member states have voluntarily suspended the use of all growth promoters irrespective of their human health importance. 

## 4. Microorganisms in Honey

The presence of Microorganisms in honey may influence quality and safety. The microbes found in honey and honeycomb are bacteria, molds, and yeast; they come from the bees, nectar or from external sources. Pollen, honey bee intestine, human, equipment, containers, winds, and dust are possible sources of microbial contamination. Pollen may be the original source of microbes in the intestines of honeybees. The intestine of bees contains 1% yeast, 27% Gram-positive bacteria (*Bacillus, Bacteridium, Streptococcus* and *Clostridium* spp.) and 70% Gram-negative bacteria (*Achromobacter, Citrobacter, Enterobacter, Erwinia, Escherichia coli, Flavobacterium, Klebsiella, Proteus,* and *Pseudomonas)* [65–67]. 

Due to various reasons, most bacteria and other microbes cannot grow or reproduce in honey. Honey has antimicrobial properties that prevent the growth of many microorganisms [68]. In addition, honey has a low water activity, preventing the multiplication and the survival of bacteria. However, few pathogens have been found in honey [68, 69]. Basically, microbes cannot replicate in honey and existence of high numbers of vegetative bacteria might be due to recent contamination. Study showed that various bacteria inoculated into aseptically collected honey held at 20°C loss viability within 8–24 days [70]. However, spore forming microorganisms can survive in honey at low temperature. *Bacillus cereus, Clostridium perfringens,* and *Clostridium botulinum* spores were inoculated into honey and stored at 25°C. The *Clostridium botulinum* population did not change over a year at 4°C.

Honey contamination with spores of *Clostridium* has been documented in many countries. Many spores of *Clostridium botulinum* type F were detected in different containers of honey products [71]. No difference in pH, hydroxymethyl furfural contents, or diastase activity was found between contaminated honey and sterilized honey. *B. alvei* stimulated the toxin production by *Clostridium botulinum* type F and therefore the growth of *Clostridium botulinum* in the honey might have been attributed to the possible stimulation by *B. alvei* [71]. 

In Argentina, *Clostridium botulinum* type A was detected in 2/177 honey samples of rural producer [72]. In Brazil, 6/85 honey samples analyzed were found positive for *Clostridium botulinum* (7.06%) and identified as producers of type A, B, and D toxins [73].

In California, methods for the isolation of *Clostridium botulinum* from honey samples are described in 1979. A total of 9/90 honey samples were positive for *Clostridium botulinum*; 6 of the positive samples had been fed to babies who developed infant botulism [74]. 

In Japan, it was found that type A, B, and C *Clostridium* spores were detected in 3/56 samples of sugar for apiculture. Type A spores were detected in some samples of raw sugar and molasses and also in 2/41 samples of brown sugar lump [75]. The existence of such contamination may contribute to contamination of honey with the spores. 

In Finland, spores of *Clostridium botulinum* were detected in 8 (7%) of the 114 Finnish and in 12 (16%) of the 76 imported honey samples [76].

Infant botulism, first described in 1976, is the most common form of botulism. It is caused by the ingestion of spores of *Clostridium botulinum*. Ingested spores multiply and produce botulinum toxin in the digestive tract of newborns and infants. A single French case was described in the literature prior to 1991. Seven cases of infant botulism identified in France between 1991 and 2009 were reported [77]. In Italy one case of infant botulism was reported due to honey [78]. A three-month-old boy of Norwegian origin who had been fed Argentinian honey developed symptoms of botulism [79]. 

In California, *Clostridium botulinum* organisms were identified in six different honey specimens fed to three patients with infant botulism. In the same city, 29.2% (12/41) of hospitalized patients had been fed honey prior to onset of constipation and, worldwide, honey exposure occurred in 34.7% (28/75) of hospitalized cases [80]. Honey consumption was associated with 15% of the reported cases of infant botulism to the Centers for Disease Control and Prevention [81]. Up to 25% of the honey products in the US contain spores of *Clostridium botulinum* [82, 83].

Hazard Analysis Critical Control Point system serves as a useful tool for beekeepers, food business operators, veterinary advisors, and for food and veterinary official control bodies in their planning and conducting of audits and for establishing priorities for the evaluation of training programs in the apicultural sector. European legislation makes many references to the key role of primary production in food safety management and the Hazard Analysis Critical Control Point System has been indicated as the preferred tool to ensure that consumers are provided with safe foods [84]. In managing risks associated with the human consumption of honey, all sectors of the production chain must be considered, including the primary production phase. 

Recently, a scientific committee of the European Union has examined the hazard of *Clostridium botulinum* in honey (EC, 2002). It has concluded that no microbiological examinations of honey are necessary, as the incidence of *Clostridium botulinum *is relatively low and that tests will not prevent infant botulism [85].

Honey used for clinical practice can be sterilized with gamma radiation to reduce the risk of botulinum spores or other possible contaminants, and gamma radiation does not affect honey's antibacterial activity [86, 87]. 

## 5. Toxic Honey

Honey produced from flowers of certain plants can cause honey intoxication and various symptoms such as dizziness, weakness, sweating, nausea, vomiting, hypotension, shock, and arrhythmia and death might be encountered. Basically, some substances are toxic to humans but are not toxic to bees [88]. Nectar of certain plants produced toxic and sometimes fatal honey (Table 2). Fermented honey produces ethanol which is toxic [89]. Indeed, there are many reports discussing toxic honey, but few observational studies in scientific literature have been reported so far [90–96]. In addition, no attempt was done to study honey toxicity in deep.

Mad honey (deli bal in Turkish) has been a topic of interest after publishing cases of honey toxicity due to ingestion of honey contaminated by Rhododendron nectar grayanotoxins [90]. This intoxication dates back to 401 BC and it is common in Turkey. Its symptoms are various from mild to severe and life threatening. In mild case, nausea, vomiting, excessive salivations, and dizziness might be encountered and in severe form cardiac complications such as complete heart block might be encountered. The main reason for toxicity is grayanotoxins, also known as andromedotoxins which are produced by plants of the *Ericaecae* family. These lead to cardiac toxicity by augmenting sodium channel permeability and vagus nerve activation. Okuyan et al. from Turkey reported 42 patients who had been hospitalized with diagnosis of “mad honey” intoxication [91]. The patients had nausea, vomiting, dizziness, fainting, and sweating, syncope (5 patients), sinus bradycardia (18 patients), complete atrioventricular block (15 patients), and nodal rhythm (9 patients). All were treated successfully. Another study from Turkey reported a retrospective case series of 19 patients admitted in 2002, poisoned by “mad” honey; the patients had nausea, vomiting, sweating, dizziness, and weakness, several hours after ingesting “mad honey” [95]. Few of the patients had hypertension and cardiac conduction abnormalities which have been resolved with atropine treatment.

## 6. Conclusion

Honey is a natural product that is widely used for both nutritional and medicinal purposes. Honey like other foods is prone to various types of contaminations and adulterations. Markets are full of unlabeled and adulterated honeys. Microbial and nonmicrobial contaminants which include pesticides, herbicides, antibiotics, or heavy metals have been reported in various honey samples all over the world. Therefore, its ingestion without knowing its source and safety might carry significant health hazards. Labeling of honey must be supported by analysis that confirms its provenance and safety. Health authorities in all nations have to introduce firm legislations and laws that control and regulate honey production, handling, and analysis to ascertain its safety. Raw honey that was not subjected for analysis or sterilization should not be used in infants. Furthermore, raw honey should not be applied to wounds or lesions without sterilization to be sure that it is safe and it also should be subjected for analysis to identity any adulteration that certainly affects its therapeutic properties. These recommendations should also be considered when other bee products such as wax, bee venom, pollen, and royal jelly are used either as dietary supplements or as medicinal remedies. Residual levels of contaminants cannot be changed through various production techniques; therefore, adequate monitoring is required. The market competition on these products imposes extra conditions that can only be ensured by complying with quality assurance and certification protocols and legislation.

## Figures and Tables

**Table 1:** Types of honey's contamination.

(A) The environmental contaminanants	
(1) Heavy metals such as lead, cadmium, and mercury	
(2) Radioactive isotopes	
(3) Organic pollutants, polychlorinated biphenyls (PCB's)	
(4) Pesticides (insecticides, fungicides, herbicides, and bactericides)	
(5) Pathogenic bacteria	
(6) Genetically modified organisms	
(B) The beekeeping contaminants	
(1) Acaricides: lipophilic synthetic compounds and nontoxic substances such as organic acids and components of essential oils	
(2) Antibiotics used for the control of bee brood diseases, mainly tetracyclines, streptomycin, sulfonamides, and chloramphenicol.	
(3) Paradichlorobenzene, used for the control of wax moth and chemical repellents	

**Table 2:** Names of some plants whose nectar gives rise to toxic honey.

*Rhododendron ponticum* (*Azalea pontica*) contains alkaloids that are poisonous to humans	
*Andromeda* flowers contain grayanotoxins which are psychoactive and toxic to humans (paralyze limbs and diaphragm and result in death)	
*Kalmia latifolia*, the calico bush, mountain laurel, or spoon wood of the northern US, and allied species produce sickness or death	
Wharangi bush, *Melicopeternata*, in New Zealand, produces toxic fatal honey	
*Datura* plants in Mexico and Hungary	
*Belladonna* flowers, henbane (Hyoscamusniger) plants in Hungary,	
*Serjanialethalis* in Brazil	
*Gelsemium sempervirens* in the American Southwest	
*Tutu* (*Coriariaarborea*), in New Zealand, produce tutin which is a member of the picrotoxin group of poisons	
*Oleander* in Mediterranean region	

## References

[1] Greenwood D (1995). Sixty years on: antimicrobial drug resistance comes of age. *The Lancet*.

[2] Ankra-Badu GA (1992). Sickle cell leg ulcers in Ghana. *East African Medical Journal*.

[3] Obi CL, Ugoji EO, Edun SA, Lawal SF, Anyiwo CE (1994). The antibacterial effect of honey on diarrhoea causing bacterial agents isolated in Lagos, Nigeria. *African Journal of Medicine and Medical Sciences*.

[4] Imperato PJ, Traoré D (1969). Traditional beliefs about measles and its treatment among the bambara of Mali. *Tropical and Geographical Medicine*.

[5] Chen L, Mehta A, Berenbaum M, Zangerl AR, Engeseth NJ (2000). Honeys from different floral sources as inhibitors of enzymatic browning in fruit and vegetable homogenates. *Journal of Agricultural and Food Chemistry*.

[6] Mundo MA, Padilla-Zakour OI, Worobo RW (2004). Growth inhibition of foodborne pathogens and food spoilage organisms by select raw honeys. *International Journal of Food Microbiology*.

[7] Al-Waili NS, Saloom KY (1999). Effects of topical honey on post-operative wound infections due to gram positive and gram negative bacteria following caesarean sections and hysterectomies. *European Journal of Medical Research*.

[8] Al-Waili NS, Salom K, Butler G, Al Ghamdi AA (2011). Honey and microbial infections: a review supporting the use of honey for microbial control. *Journal of Medicinal Food*.

[9] Al-Waili NS, Salom K, Al-Ghamdi AA (2011). Honey for wound healing, ulcers, and burns; data supporting its use in clinical practice. *The Scientific World Journal*.

[10] Othman NH (2012). Honey and cancer: sustainable inverse relationship particularly for developing nations-a review. *Evidence-Based Complementary and Alternative Medicine*.

[11] Parmar J, Hunjan P, Brown A, Telfer M Honey dressing use for the management of split thickness skin graft donor sites: a technical note.

[12] Kelly S, Heaton K, Hoogewerff J (2005). Tracing the geographical origin of food: the application of multi-element and multi-isotope analysis. *Trends in Food Science and Technology*.

[13] Directive

[14] Tillotson GS, Doern GV, Blondeau JM (2006). Optimal antimicrobial therapy: the balance of potency and exposure. *Expert Opinion on Investigational Drugs*.

[15] Korta E, Bakkali A, Berrueta LA, Gallo B, Vicente F (2002). Study of an accelerated solvent extraction procedure for the determination of acaricide residues in honey by high-performance liquid chromatography-diode array detector. *Journal of Food Protection*.

[16] Mullin CA, Frazier M, Frazier JL (2010). High levels of miticides and agrochemicals in North American apiaries: implications for honey bee health. *PloS ONE*.

[17] Frazier M, Mullin C, Frazier J, Ashcraft S (2008). What have pesticides got to do with it?. *American Bee Journal*.

[18] Martel AC, Zeggane S, Aurières C, Drajnudel P, Faucon JP, Aubert M (2007). Acaricide residues in honey and wax after treatment of honey bee colonies with Apivar or Asuntol 50. *Apidologie*.

[19] Bogdanov S (2006). Contaminants of bee products. *Apidologie*.

[20] Codex Alimentarius

[22] Food and Drug Administration of the United States http://www.cfsan.fda.gov/.

[23] Wallner K (1999). Varroacides and their residues in bee products. *Apidologie*.

[24] Menkissoglu-Spiroudi U, Tsigouri AD, Diamantidis GC, Thrasyvoulou AT (2001). Residues in honey and beeswax caused by beekeeping treatments. *Fresenius Environmental Bulletin*.

[25] Beliën T, Kellers J, Heylen K (2009). Effects of sublethal doses of crop protection agents on honey bee (*Apis mellifera*) global colony vitality and its potential link with aberrant foraging activity. *Communications in Agricultural and Applied Biological Sciences*.

[26] Choudhary A, Sharma DC (2008). Pesticide residues in honey samples from himachal pradesh (India). *Bulletin of Environmental Contamination and Toxicology*.

[27] Anju R, Beena K, Gahlawat SK, Sihag RC, Kathpal TS (1997). Multiresidue analysis of market honey samples for pesticidal contamination. *Pesticide Research Journal*.

[28] Al-Rifai J, Akeel N (1997). Determination of pesticide residues in imported and locally produced honey in Jordan. *Journal of Apicultural Research*.

[29] Yavuz H, Guler GO, Aktumsek A, Cakmak YS, Ozparlak H (2010). Determination of some organochlorine pesticide residues in honeys from Konya, Turkey. *Environmental Monitoring and Assessment*.

[30] Erdoğrul Ö (2007). Levels of selected pesticides in honey samples from Kahramanmaraş, Turkey. *Food Control*.

[31] Das YK, Kaya S (2009). Organophosphorus insecticide residues in honey produced in Turkey. *Bulletin of Environmental Contamination and Toxicology*.

[32] Beyoğlu D, Omurtag GZ (2007). Occurrence of naphthalene in honey consumed in Turkey as determined by high-pressure liquid chromatography. *Journal of Food Protection*.

[33] Adamczyk S, Lázaro R, Pérez-Arquillué C, Conchello P, Herrera A (2005). Evaluation of residues of essential oil components in honey after different anti-varroa treatments. *Journal of Agricultural and Food Chemistry*.

[34] Blasco C, Fernández M, Pena A (2003). Assessment of pesticide residues in honey samples from Portugal and Spain. *Journal of Agricultural and Food Chemistry*.

[35] Chauzat MP, Faucon JP (2007). Pesticide residues in beeswax samples collected from honey bee colonies (*Apis mellifera* L.) in France. *Pest Management Science*.

[36] Rexilius L (1986). Rückstände von Pflanzenbehandlungsmitteln in Rapshonig der Ernte 1984 aus Schleswig-Holstein—EineStatusuntersuchung. *Nachrichtenbl Dtsch Pflanzenschutzd*.

[37] Fléché C, Clément M, Zeggane S, Faucon J (1997). Contamination des produits de la ruche et risques pour la santé humaine: situation en France. *Revue Scientifique et Technique (International Office of Epizootics)*.

[38] Rüegg J (1995). MoniliaimObstbau—Prüfung von Fungiziden in der biologi schenind Integrierten Produktion. *Obst-und Weinbau*.

[39] Büchler R, Volkmann B (2003). Rückstände von Carbendazim und anderen Fungizidenim Bienenhonigaufgrund der Blütespritzung von Winterraps. *Gesun de Pflanzen*.

[40] Fell RD, Cobb JM (2009). Miticide residues in Virginia honeys. *Bulletin of Environmental Contamination and Toxicology*.

[41] Jan J, Cerne K (1993). Distribution of some organochlorine compounds (PCB, CBz, and DDE) in beeswax and honey. *Bulletin of Environmental Contamination and Toxicology*.

[42] Morse RA, Culliney TW, Gutenmann WH, Littman C, Lisk D (1987). Polychlorinated biphenyls in honey bees. *Bulletin of Environmental Contamination and Toxicology*.

[43] Anderson J, Wojtas M (1986). Honey bees (Hymenoptera: Apidae) contaminated with pesticides and polychlorinated biphenyls. *Journal of Economic Entomology*.

[44] Lorenz ES (2009). Potential health effects of pesticides. *Ag Communications and Marketing*.

[45] Ritter L, Solomon KR, Forget J, Stemeroff M, O'Leary C

[46] Lim S, Cho YM, Park KS, Lee HK (2010). Persistent organic pollutants, mitochondrial dysfunction, and metabolic syndrome. *Annals of the New York Academy of Sciences*.

[47] Centers for Disease Control and Prevention

[48] Ortelli D, Edder P, Corvi C (2004). Analysis of chloramphenicol residues in honey by liquid chromatography-tandem mass spectrometry. *Chromatographia*.

[49] Saridaki-Papakonstadinou M, Andredakis S, Burriel A, Tsachev I (2006). Determination of tetracycline residues in Greek honey. *Trakia Journal of Sciences*.

[50] Granja R, Niño AM, Zucchetti R, Niño RM, Patel R, Salerno AG (2009). Determination of erythromycin and lyiosin residues in honey by LC/MS/MS. *Journal of AOAC International*.

[51] Adams SJ, Fussell RJ, Dickinson M, Wilkins S, Sharman M (2009). Study of the depletion of lincomycin residues in honey extracted from treated honeybee (*Apis mellifera* L.) colonies and the effect of the shook swarm procedure. *Analytica Chimica Acta*.

[52] Chen TB, Deng WH, Lu WH, Chen RM, Rao PF (2001). Detection of residual antibiotics in honey with capillary electrophoresis. *School Equipment Production Unit*.

[53] Johnson S, Jadon N http://www.scribd.com/doc/38591126/7/Review-of-Literature.

[54] Solomon R, Satheeja S, Vimalan J (2006). Prevalence of antibiotics in nectar and honey in South Tamil Nadu, India. *Integra Biosciences*.

[55] Forsgren E (2010). European foulbrood in honey bees. *Journal of Invertebrate Pathology*.

[56] Martel AC, Zeggane S, Drajnudel P, Faucon JP, Aubert M (2006). Tetracycline residues in honey after hive treatment. *Food Additives and Contaminants*.

[57] Thompson HM, Waite RJ, Wilkins S (2005). Effects of european foulbrood treatment regime on oxytetracycline levels in honey extracted from treated honeybee (*Apis mellifera*) colonies and toxicity to brood. *Food Additives and Contaminants*.

[58] Kaufmann A, Kaenzig A (2004). Contamination of honey by the herbicide asulam and its antibacterial active metabolite sulfanilamide. *Food Additives and Contaminants*.

[59] Vidal JLM, Aguilera-Luiz MDM, Romero-González R, Frenich AG (2009). Multiclass analysis of antibiotic residues in honey by ultraperformance liquid chromatography-tandem mass spectrometry. *Journal of Agricultural and Food Chemistry*.

[60] Brasse D (2001). Stellungnahme der BBA zum Streptomycin-Problem. Teil 2: Bewertung der RückstandswerteimHonig. *Allgemeine Deutsche Imkerzeitung*.

[61] Reybroeck W (2003). Residues of antibiotics and sulfonamides in honey on the Belgian market. *Apiacta*.

[62] Diserens J Contaminants and residues in Food. Strategies (if any) to screen and analyze veterinary drug residues in food from animal origin. http://www.biocop.org/.../ContaminantsResiduesinFood5thFresenuisppt.pdf.

[63] Velicer CM, Heckbert SR, Lampe JW, Potter JD, Robertson CA, Taplin SH (2004). Antibiotic use in relation to the risk of breast cancer. *The Journal of the American Medical Association*.

[64] Paige J, Tollefson L, Miller M (1997). Public health impact on drug residues in animal tisues. *Veterinary and Human Toxicology*.

[65] Gilliam M, Prest DB (1987). Microbiology of feces of the larval honey bee, *Apis mellifera*. *Journal of Invertebrate Pathology*.

[66] El-leithy M, El-sibael K (1992). Role of microorganisms isolated from bees, its ripening and fermentation of honey. *Egyptian Journal of Microbiology*.

[67] Tysset C, De Rantline de la Roy Durand C (1991). *Contribution to the Study of Intestinal Microbial Infection of Healthy Honeybees: Inventory of Bacterial Population by Negative Organisms*.

[68] Snowdon JA, Cliver DO (1996). Microorganisms in honey. *International Journal of Food Microbiology*.

[69] Snowdon J (1999). The microbiology of honey—meeting your buyers' specifications. *American Bee Journal*.

[70] Olaitan PB, Adeleke OE, Ola IO (2007). Honey: a reservoir for microorganisms and an inhibitory agent for microbes. *African Health Sciences*.

[71] Nakano H, Sakaguchi G (1991). An unusually heavy contamination of honey products by *Clostridium botulinum* type F and *Bacillus alvei*. *FEMS Microbiology Letters*.

[72] de Centorbi OP, Satorres SE, Alcaraz LE, Centorbi HJ, Fernández R (1997). Detection of *Clostridium botulinum* spores in honey. *Revista Argentina de microbiología*.

[73] Schocken-Iturrino RP, Carneiro MC, Kato E, Sorbara JOB, Rossi OD, Gerbasi LER (1999). Study of the presence of the spores of *Clostridium botulinum* in honey in Brazil. *FEMS Immunology and Medical Microbiology*.

[74] Midura TF, Snowden S, Wood RM, Arnon SS (1979). Isolation of *Clostridium botulinum* from honey. *Journal of Clinical Microbiology*.

[75] Nakano H, Yoshikuni Y, Hashimoto H, Sakaguchi G (1992). Detection of *Clostridium botulinum* in natural sweetening. *International Journal of Food Microbiology*.

[76] Nevas M, Hielm S, Lindström M, Horn H, Koivulehto K, Korkeala H (2002). High prevalence of *Clostridium botulinum* types A and B in honey samples detected by polymerase chain reaction. *International Journal of Food Microbiology*.

[77] King LA, Popoff MR, Mazuet C, Espié E, Vaillant V, de Valk H (2010). Infant botulism in France. *Archives de Pediatrie*.

[78] Fenicia L, Ferrini AM, Aureli P, Pocecco M (1993). A case of infant botulism associated with honey feeding in italy. *European Journal of Epidemiology*.

[79] Tølløfsrud PA, Kvittingen EA, Granum PE, Vøllo A (1998). Botulism in newborn infants. *Tidsskrift for den Norske Laegeforening*.

[80] Arnon SS, Midura TF, Damus K, Thompson B, Wood R, Chin J (1979). Honey and other environmental risk factors for infant botulism. *Journal of Pediatrics*.

[81] Midura TF (1996). Update: infant botulism. *Clinical Microbiology Reviews*.

[82] Cox N, Hinkle R (2002). Infant botulism. *American Family Physician*.

[83] Gimenez JA, Gimenez MA, Dasgupta BR (1991). Characterization of the neurotoxin isolated from a *Clostridium baratii* strain implicated in infant botulism. *Infection and Immunity*.

[84] Formato G, Zilli R, Condoleo R, Marozzi S, Davis I, Smulders F (2011). Risk management in primary apicultural production. part 2: a hazard analysis critical control point approach to assuring the safety of unprocessed honey. *The Veterinary Quarterly*.

[85] Bogdanov S, Jurendic T, Sieber R, Gallmann P (2008). Honey for nutrition and health: a review. *Journal of the American College of Nutrition*.

[86] Postmes T, van den Bogaard AE, Hazen M (1995). The sterilization of honey with cobalt 60 gamma radiation: a study of honey spiked with spores of *Clostridium botulinum* and *Bacillus subtilis*. *Experientia*.

[87] Molan PC, Allen KL (1996). The effect of gamma-irradiation on the antibacterial activity of honey. *Journal of Pharmacy and Pharmacology*.

[88] Adrienne M (1995). Mad honey, toxic honey in history. *Archaeology*.

[89] Kettlewellh B (1945). A story of nature's Debauch. *Entomologist*.

[90] Koca I, Koca AF (2007). Poisoning by mad honey: a brief review. *Food and Chemical Toxicology*.

[91] Okuyan E, Uslu A, Ozan Levent M (2010). Cardiac effects of “mad honey”: a case series. *Clinical Toxicology*.

[92] Daugherty CG (2005). Toxic honey and the march up-country. *Journal of Medical Biography*.

[93] Geroulanos S, Attinger B, Cakmakçi M (1992). Honey-induced poisoning. *Schweizerische Rundschau fur Medizin Praxis*.

[94] Marciniak J, Sikorski M (1972). Intoxication with alcaloids of datura stramonium and datura inoxia following honey ingestion. *Polski Tygodnik Lekarski*.

[95] Özhan H, Akdemir R, Yazici M, Gündüz H, Duran S, Uyan C (2004). Cardiac emergencies caused by honey ingestion: a single centre experience. *Emergency Medicine Journal*.

[96] http://en.wikipedia.org/wiki/Bees_and_toxic_chemicals.

